# ER-Mitochondria Calcium Flux by β-Sitosterol Promotes Cell Death in Ovarian Cancer

**DOI:** 10.3390/antiox10101583

**Published:** 2021-10-08

**Authors:** Hyocheol Bae, Sunwoo Park, Jiyeon Ham, Jisoo Song, Taeyeon Hong, Jin-Hee Choi, Gwonhwa Song, Whasun Lim

**Affiliations:** 1 Department of Oriental Biotechnology, College of Life Sciences, Kyung Hee University, Yongin-si 17104, Korea; bhc7@khu.ac.kr; 2 Department of Plant & Biomaterials Science, Gyeongsang National University, Jinju-si 52725, Korea; sw.park@gnu.ac.kr; 3 Department of Biotechnology, College of Life Sciences and Biotechnology, Korea University, Seoul 02841, Korea; glorijy76@korea.ac.kr; 4 Department of Food and Nutrition, College of Science and Technology, Kookmin University, Seoul 02707, Korea; js_song97@kookmin.ac.kr (J.S.); taeyeon97@kookmin.ac.kr (T.H.); 5 Department Food Service Management and Nutrition, Kongju National University, Yesan 32439, Korea; second86@kongju.ac.kr

**Keywords:** β-sitosterol, ovarian cancer, ER-mitochondria calcium flux, apoptosis, 3D spheroid

## Abstract

Phytosterols, which are derived from plants, have various beneficial physiological effects, including anti-hypercholesterolemic, anti-inflammatory, and antifungal activities. The anticancer activities of natural products have attracted great attention, being associated with a low risk of side effects and not inducing antineoplastic resistance. β-sitosterol, a phytosterol, has been reported to have anticancer effects against fibrosarcoma and colon, breast, lung, and prostate cancer. However, there are no reports of its activity against ovarian cancer. Therefore, we investigated whether β-sitosterol shows anticancer effects against ovarian cancer using human ovarian cancer cell lines. We confirmed that β-sitosterol induced the apoptosis of ovarian cancer cells and suppressed their proliferation. It triggered pro-apoptosis signals and the loss of mitochondrial membrane potential, enhanced the generation of reactive oxygen species and calcium influx through the endoplasmic reticulum-mitochondria axis, and altered signaling pathways in human ovarian cancer cells. In addition, we observed inhibition of cell aggregation, suppression of cell growth, and decreased cell migration in ovarian cancer cells treated with β-sitosterol. Further, our data obtained using ovarian cancer cells showed that, in combination with standard anti-cancer drugs, β-sitosterol demonstrated synergistic anti-cancer effects. Thus, our study suggests that β-sitosterol may exert anti-cancer effects against ovarian cancer in humans.

## 1. Introduction

Ovarian cancer originates in the ovary and invades the surrounding tissues, including the abdomen, lymph nodes, lungs, and liver. Ovarian cancer is difficult to diagnose because in the early stages there are no visible symptoms, and only about 20% of ovarian cancers are detected early [1]. The overall five-year survival rate for ovarian cancer patients is 45% in the USA [2]. Therefore, there is a need for novel anticancer drugs without antineoplastic resistance or side effects to treat this condition. Ovarian cancer is a heterogeneous disease that includes several epithelial varieties with morphological and molecular subtypes, making effective treatments for specific types a necessity [3]. Ovarian cancer is divided into five categories: high- and low-grade serous, clear cell, endometrioid, and mucinous. Of these, high-grade serous and clear cell carcinomas are the most common and the first and second most prevalent subtypes in North America, respectively [4,5]. In this study, we used two types of ovarian cancer cell lines representative of clear cell carcinoma (ES2) and high-grade serous adenocarcinoma (OV90).

β-sitosterol is a compound found in a diverse range of plant materials, including vegetable oils, nuts, and avocados. It is an example of a phytosterol and is reported to have various physiological activities, such as anti-hypercholesterolemic, anti-inflammatory, antibacterial, and antifungal properties [6]. Its structure is similar to that of cholesterol, with an additional ethyl group at C-24. In addition, β-sitosterol has therapeutic effect against benign prostatic hyperplasia (BPH) [7,8]. Phytosterols are generally known to be beneficial, but excessive absorption of vegetable sterols can cause phytosterolemia [9]. In the field of cancer research, β-sitosterol has been reported to have anticancer effects on various types of cancer, including fibrosarcoma [10] and colon [8,11], breast [12,13], and prostate cancer [14]. In addition, β-sitosterol was also shown to enhance the effect of tamoxifen (TAM), a selective estrogen receptor modulator, in the treatment of breast cancer [15]. Few studies have been conducted on the anticancer effects of β-sitosterol in human ovarian cancer [16]. However, previous studies have focused on case-control clinical studies rather than on the mode of action of β-sitosterol in ovarian cancer. Therefore, we sought to identify whether β-sitosterol could inhibit the development of ovarian cancer using human ovarian cancer cell lines.

## 2. Materials and Methods

### 2.1. Reagents

β-Sitosterol (cat number: S9889), *cis*-diammineplatinum (II) dichloride (cisplatin), and paclitaxel were purchased from Sigma-Aldrich (St. Louis, MO, USA).

### 2.2. Cell Incubation

Ovarian cancer cells were purchased from the American Type Culture Collection (ATCC; Manassas, VA, USA). Both lines of ovarian cancer cells were grown in a cell incubator at 37 °C with 5% carbon dioxide (CO_2_), in McCoy’s 5A medium supplemented with 10% fetal bovine albumin (FBS). Prior to the tests, all cells were starved in a serum-free medium for 24 h.

### 2.3. Western Blot

ES2 and OV90 cells were treated with β-sitosterol (0, 10, 25, and 50 µg/mL) for 24 h. The cells were washed twice with 0.01% calcium ion and 0.01% magnesium ion dissolved in phosphate buffer saline (PBS) before protein extraction. The cells were subsequently detached and lysed using the Triton-X lysis buffer. The concentration of the extracted proteins was measured using the Bradford assay [17]. Western blotting was performed using the extracted proteins from β-sitosterol-treated ovarian cancer cells. All antibodies for each signal protein used in this study were the same as those used in a previous study [18].

### 2.4. Annexin V and PI Staining

Cells from both cell line were incubated in a cell culture plate and starved for one day. They were treated with β-sitosterol at different concentration (0, 10, 25, and 50 µg/mL) for 48 h. All cells were rinsed twice and detached using 0.25% trypsin-EDTA (ethylenediamine-N,N,N′,N′-tetraacetic acid, edetic acid) and subsequently pelleted by centrifugation at 1250 rpm for 5 min. Each prepared cell sample was incubated with FITC Annexin V (5 µL) and propidium iodide (PI; 5 μL) in the dark for 15 min at room temperature. Cell apoptosis was observed using a flow cytometer (BD Bioscience, Franklin Lakes, NJ, USA).

### 2.5. JC-1 Staining

ES2 and OV90 cells were maintained at 50% confluence in 6-well plates. FBS-starved cells were incubated with β-sitosterol (0, 10, 25, and 50 µg/mL) for 48 h. Cells were washed twice to remove β-sitosterol before the addition of 0.25% trypsin-EDTA. Suspended cells were collected via centrifugation at 1250 rpm for 5 min and washed twice using PBS to remove trypsin-EDTA. They were then incubated with membrane-permeable 5,5,6,6′-tetrachloro-1,1′,3,3′-tetraethyl-imidacarbocyanine iodide (JC-1) (Sigma-Aldrich) for 20 min at 37 °C and analyzed according to the manufacturer’s instructions. When mitochondria maintain their normal mitochondrial membrane potential (MMP), JC-1 enters the electron transport system and forms J-aggregates, exhibiting red fluorescence. In contrast, if the mitochondria lack MMP, JC-1 forms monomers and remains in the cytosol, exhibiting green fluorescence. This difference allows JC-1 to detect MMP changes in cells [19].

### 2.6. Measurement of ROS Production

The ovarian cancer cells were incubated in 6-well plates and then detached with 0.25% trypsin-EDTA. Both cell types were washed twice with 2,7-dichlorofluorescein diacetate (DCFH-DA; Sigma, 10 µM) for 30 min and subsequently treated with β-sitosterol (0, 10, 25, and 50 µg/mL) for 1 h. The amount of accumulated reactive oxygen species (ROS) throughout β-sitosterol incubation was determined using a flow cytometer (BD Bioscience). DCFH-DA can be oxidized by the major physiological ROS produced by cellular metabolism, including superoxide anions, hydroxyl radicals, and hydrogen peroxide. Therefore, the oxidation of DCFH-DA to 2′,7′-dichlorofluorescein (DCF) has been used extensively as an indicator for the detection of total ROS, including hydroxyl radicals (OH) and nitrogen dioxide (NO_2_) [20].

### 2.7. Cytosolic Ca^2+^ Level Analysis

Cell from the two cell lines were grown and starved in FBS or non-FBS medium. Both cell cultures were treated with β-sitosterol (0, 10, 25, and 50 µg/mL) for 48 h. Cells were collected and rinsed twice with PBS. Prepared cells were stained with fluo-4 acetoxymethyl ester (AM; 3 μM) (Invitrogen) for 20 min at 37 °C. Fluo-4 is a molecule that enhances fluorescence when binding to calcium and is an analogue of fluo-3 with two chlorine substituents instead of fluorine, providing a high fluorescence signal at 488 nm [21]. The 488 nm green fluorescence of Fluo-4 was observed using a flow cytometer (BD Bioscience).

### 2.8. Mitochondrial Ca^2+^ Level Analysis

The ovarian cancer cells were grown and starved in FBS or non-FBS medium. The starved cells were treated with β-sitosterol (0, 10, 25, and 50 µg/mL) for 48 h and detached with 0.25% trypsin-EDTA, subsequently being rinsed twice with PBS and stained with rhod-2 acetoxymethyl (AM) for 20 min at 37 °C. Changes in fluorescence were observed using flow cytometry (BD Bioscience). Rhod-2 AM is an AM ester of the red fluorescent mitochondrial calcium indicator. The AM group facilitates the compound’s cellular uptake and is removed by cytoplasmic esterases, resulting in intracellular accumulation of rhod-2. Rhod-2 AM selectively accumulates within mitochondria and, consequently, finds frequent use in monitoring calcium changes within these organelles. The excitation and emission maxima were 557 and 581 nm, respectively [22,23,24].

### 2.9. 3D Cell Culture Assay

Cells were cultured by the hanging drop method, with cells hanging from the cover of the culture dish (3 × 10^3^ cells/drop). Vehicle-treated and β-sitosterol (50 µg/mL)-treated cells were cultured for 72 h. Cancer cell morphology was investigated with a DM3000 microscope (Leica, Wetzlar, Germany). Tumor area was calculated using ImageJ software (http://rsb.info.nih.gov/ij/docs/index.html, accessed on 21 May 2020), while 3D density was estimated by using the ReViSP software (https://sourceforge.net/projects/revisp/, accessed on 21 May 2020).

### 2.10. Proliferation Assay

Proliferating ovarian cancer cells were incubated with β-sitosterol (0, 10, 25, and 50 µg/mL) for 48 h with and without 20 μM cisplatin and with 20 μM paclitaxel, cisplatin, or paclitaxel alone. Proliferation was detected using ELISA, a BrdU Kit (Roche, Basel, Switzerland), and a microplate reader (BioTek, Winooski, VT, USA) according to the manufacturer’s instructions.

### 2.11. Migration Assay

Cells were incubated on Transwell inserts with and without β-sitosterol treatment for 12 h. The cells that located on the Transwell membranes were fixed with methanol for 10 min, prior to being stained with hematoxylin for 30 min. The membrane was rinsed with tap water thrice, placed on slides, and covered with mounting media. Each slide was observed using a DM3000 microscope (Leica) after drying for 24 h.

### 2.12. Statistical Analysis

All quantitative data were analyzed by least-squares analysis of variance (ANOVA) using the general linear model procedure of the Statistical Analysis System (SAS) program (9.4 version, Cary, NC, USA). All experiments were performed at least thrice. Statistical significance was set at *p* < 0.05, and all significance was identified as an appropriate error term by estimating the mean square of the error.

## 3. Results

### 3.1. Activation of Cell Death Signals and Cell Apoptosis by β-Sitosterol in Human Ovarian Cancer Cells

**Figure 1:**
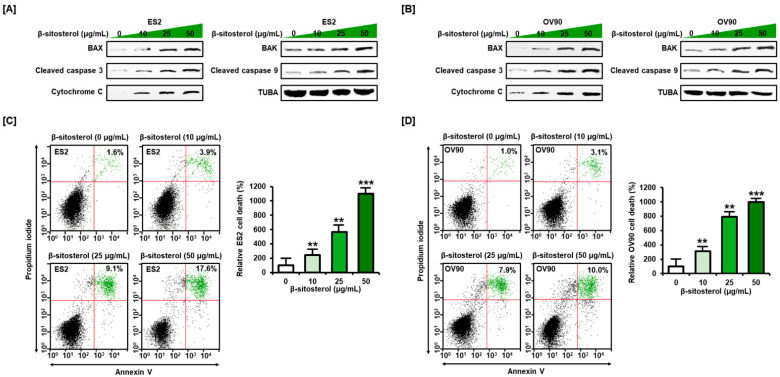
Western blot and annexin V and propidium iodide (PI) staining were performed to investigate cell death in cultures of both cell lines. (**A**,**B**) Western blot bands showing the expression of pro-apoptotic signal components in β-sitosterol (0, 10, 25, and 50 µg/mL)-treated cells. Alpha-tubulin (TUBA), used as a control, is shown at the bottom right of each figure. (**C**,**D**) Quadrants of the dot blot represent the state of cell apoptosis in ES2 (**C**) and OV90 (**D**) cells. Comparative bar graphs represent the relative change in late apoptosis following β-sitosterol treatment (0, 10, 25, and 50 µg/mL) compared to the control (100%) in ES2 and OV90 cells. The asterisks indicate significant differences between treated and control cells (*** *p* < 0.001 and ** *p* < 0.01).

β-sitosterol (0, 10, 25, and 50 µg/mL) was found to stimulate the expression of BAX, BAK, cleaved caspase 3, cleaved caspase 9, and cytochrome C in ES2 and OV90 cells in a dose-dependent manner (Figure 1A,B). The level of alpha tubulin (TUBA) was not changed by β-sitosterol. These data show that pro-apoptotic signals were increased by β-sitosterol in ovarian cancer cells. We investigated the cell state by annexin V and PI staining, to confirm the programmed cell apoptosis induced by β-sitosterol. In ES2 cells, late apoptosis (upper right quadrant) was increased to 243%, 568%, and 1100% by 10, 25, and 50 µg/mL of β-sitosterol, respectively, compared to the vehicle-treated control (100%) (Figure 1C). In OV90 cells, cell death (upper right quadrant) was increased to 310%, 790%, and 1000% by 5, 10, and 50 µg/mL of β-sitosterol, respectively, compared to the vehicle-treated control (100%), (Figure 1D).

### 3.2. Changes in Mitochondrial Membrane Potential (MMP) and ROS Levels Caused by β-Sitosterol in Ovarian Cancer Cells

**Figure 2:**
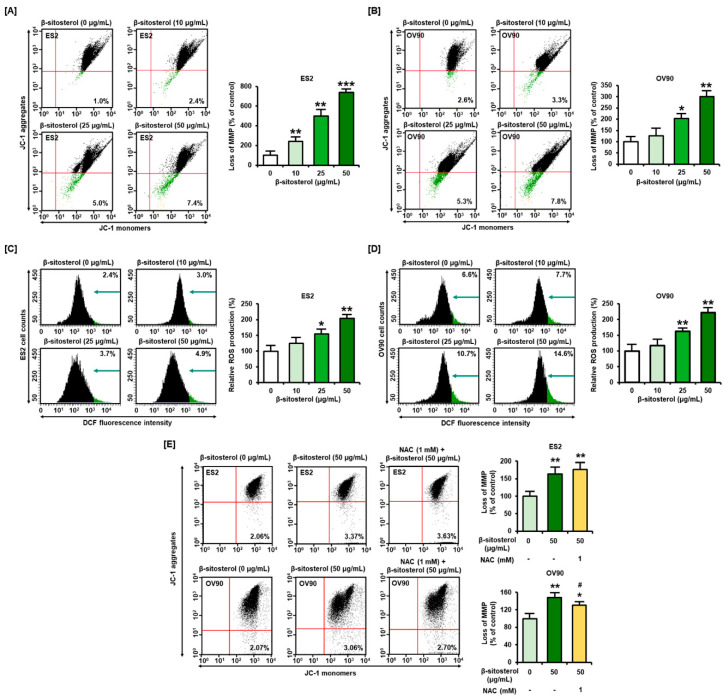
Alteration of mitochondrial membrane potential (MMP) and reactive oxygen species (ROS) generation by β-sitosterol in the two cell types. (**A**,**B**) We used 5,5′,6,6′-tetrachloro-1,1′,3,3′-tetraethyl-imidacarbocyanine iodide (JC-1) dye to investigate the alteration of MMP by β-sitosterol (0, 10, 25, and 50 µg/mL). Quadrants of the dot blot represent the state of MMP in ES2 and OV90 cells. Comparative bar graphs represent the loss of MMP compared to the control group (100%) in ES2 and OV90 cells. (**C**,**D**) DCF fluorescence intensity was analyzed to investigate the change in ROS production following β-sitosterol treatment (0, 10, 25, and 50 µg/mL). The histograms represent the state of ROS production in ES2 and OV90 cells. The comparative bar graphs represent ROS production compared to the vehicle-treated control (100%) in ES2 and OV90 cells. DCF: dichlorofluorescein. (**E**) Alteration of MMP by β-sitosterol with or without NAC (1 mM) was determined. The asterisks indicate significant differences between treated and control cells (*** *p* < 0.001, ** *p* < 0.01, and * *p* < 0.05); # indicates significant differences as compared to cells treated with β-sitosterol alone.

MMP was dramatically changed by treatment with β-sitosterol (0, 10, 25, and 50 µg/mL) in ES2 cells. In ES2 cells, an MMP loss of 240%, 500%, and 740% was observed following treatment with 10, 25, and 50 µg/mL of β-sitosterol, respectively, compared to the vehicle-treated control (100%) (Figure 2A). Similarly, in OV90 cells, an MMP loss of 127%, 204%, and 300% was observed following treatment with 10, 25, and 50 µg/mL of β-sitosterol, respectively, compared to the control (100%) (Figure 2B). ROS production in ES2 cells increased in a dose-dependent manner to 125%, 154%, and 204%, by 10, 25, and 50 µg/mL of β-sitosterol, respectively, compared to the vehicle-treated control (100%) (Figure 2C). ROS generation in OV90 cells also increased to 117%, 162%, and 221%, following treatment with 10, 25, and 50 µg/mL of β-sitosterol, respectively, compared to the control (100%) (Figure 2D). Further, we pre-treated the cells with N-acetylcysteine (NAC) for 1 h before treatment with β-sitosterol to figure out the relationship between β-sitosterol-induced mitochondrial dysfunction and ROS generation (Figure 2E). Although there were no alleviation effects of MMP loss on ES2 cells, β-sitosterol-induced MMP loss in OV90 was slightly suppressed by NAC (Figure 2E). These results might imply that β-sitosterol mainly exerted a detrimental effect in mitochondria, and as a result, ROS homeostasis was indirectly disturbed.

### 3.3. Excessive Overload of Cytosolic and Mitochondrial Calcium Levels in β-Sitosterol-Treated Ovarian Cancer Cells

**Figure 3:**
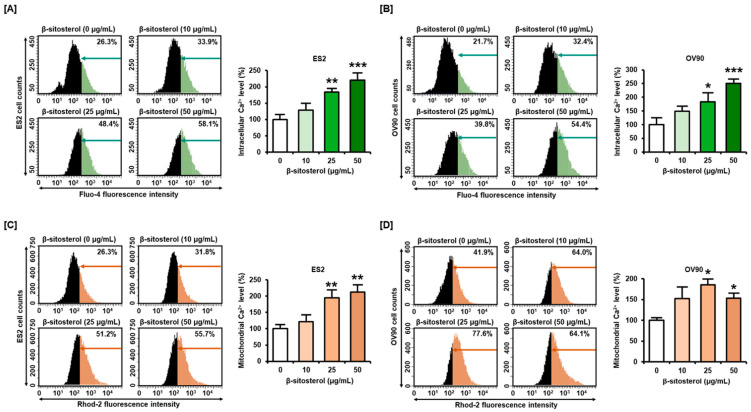
Calcium concentration in cytosol and mitochondria of β-sitosterol-treated ovarian cancer cells. (**A**,**B**) Fluo-4 dye was used to investigate the intracellular calcium levels. The histograms represent the change of cytosolic calcium by β-sitosterol (0, 10, 25, and 50 µg/mL) in ES2 and OV90. (**C**,**D**) Rhod-2 fluorescence was analyzed to investigate mitochondrial calcium levels in β-sitosterol-treated ovarian cancer cells. The histograms represent changes in mitochondrial calcium caused by β-sitosterol (0, 10, 25, and 50 µg/mL) in ES2 and OV90 cells. Comparative bar graphs represent the change of cytosolic and mitochondrial calcium levels by β-sitosterol compared to the vehicle-treated control (100%) in ES2 and OV90. The asterisks indicate significant differences between treated and control cells (*** *p* < 0.001, ** *p* < 0.01, and * *p* < 0.05).

To confirm the regulation of calcium concentrations in the cytosol and mitochondria, we incubated ES2 and OV90 cells with 0, 10, 25, and 50 µg/mL of β-sitosterol for 48 h. Then, calcium levels in the cytosol and mitochondria were analyzed by staining with fluo-4-AM and rhod-2, respectively. Cytosolic calcium levels in ES2 cells were increased to 129%, 184%, and 221% following treatment with 10, 25, and 50 µg/mL of β-sitosterol, respectively, compared to the vehicle-treated control (100%) (Figure 3A). In OV90 cells, the cytosolic calcium levels were increased to 149%, 183%, and 251%, by 10, 25, and 50 µg/mL of β-sitosterol, respectively, compared to the control (100%) (Figure 3B). In addition, the mitochondrial calcium levels in ES2 cells were increased to 195%, and 4212%, by 25, and 50 µg/mL of β-sitosterol, respectively, compared to the vehicle-treated control (100%) (Figure 3C). In OV90 cells, the mitochondrial calcium levels were increased to 153%, 185%, and 153%, by 10, 25, and 50 µg/mL of β-sitosterol, respectively, compared to the control (100%) (Figure 3D).

### 3.4. Regulation of ER Stress and of the ER-Mitochondrial Axis by β-Sitosterol in the Two Cell Lines

**Figure 4:**
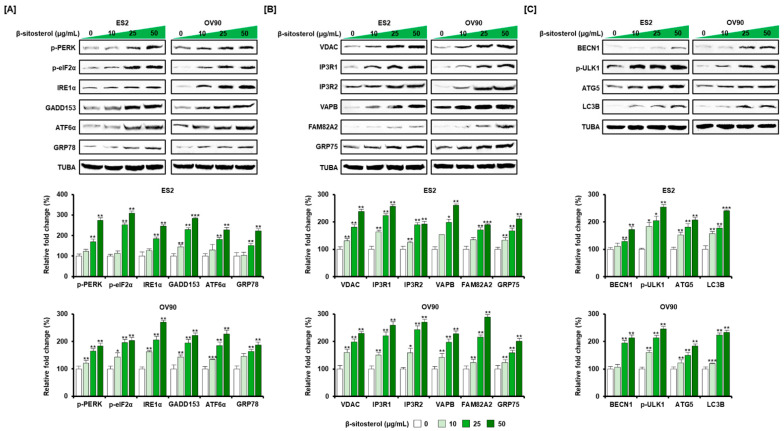
Increased expression of ER stress sensors and ER-mitochondrial axis and autophagy proteins by β-sitosterol in ovarian cancer cells. Western blot bands of UPR proteins (**A**) including protein kinase R (PKR)-like endoplasmic reticulum kinase (PERK), eukaryotic translation initiation factor 2α (eIF2α), inositol-requiring enzyme-1α (IRE1α), growth arrest and DNA damage 153 (GADD153), activating transcription factor 6α (ATF6α), 78 kDa glucose-regulated protein (GRP78), ER-mitochondrial axis proteins (**B**) voltage-dependent anion channel (VDAC), IP3 Receptor 1 (IP3R1), IP3 Receptor 2 (IP3R2), vesicle-associated membrane protein (VAPB), regulator of microtubule dynamics 3, RMDN3 (FAM82A2), glucose-regulated protein 75 (GRP75), and autophagy proteins (**C**) beclin 1 (BECN1), UNC-51-like kinase 1 (ULK1), autophagy-related 5 (ATG5), autophagy marker Light Chain 3 B (LC3B) after β-sitosterol (0, 10, 25, and 50 µg/mL) treatment. Alpha-tubulin (TUBA), used as a control, is shown at the bottom of each set. The asterisks indicate significant differences between treated and control cells (*** *p* < 0.001, ** *p* < 0.01, and * *p* < 0.05).

ER stress and the ER-mitochondrial axis were investigated using western blot analysis. Activation of a representative unfolded protein response (UPR) was observed. Phosphor-PERK (p-PERK), p-eIF2α, IRE1α, GADD153, ATF6α, GRP78 were found to be activated by β-sitosterol (0, 10, 25, and 50 µg/mL) treatment, compared to the level of TUBA in ES2 and OV90 cells (Figure 4A). ER-mitochondria tethering proteins including VDAC, IP3R1, IP3R2, VAPB, FAM82A2, and GRP75 were also activated in β-sitosterol-incubated ovarian cancer cells (Figure 4B). Under the same conditions, autophagosome proteins such as BECN1, p-ULK1, ATG5, and LC3B were induced by β-sitosterol in the examined ovarian cancer cells (Figure 4C).

### 3.5. Restriction of Cell Growth and Cell Cycle by β-Sitosterol in Ovarian Cancer Cells

**Figure 5:**
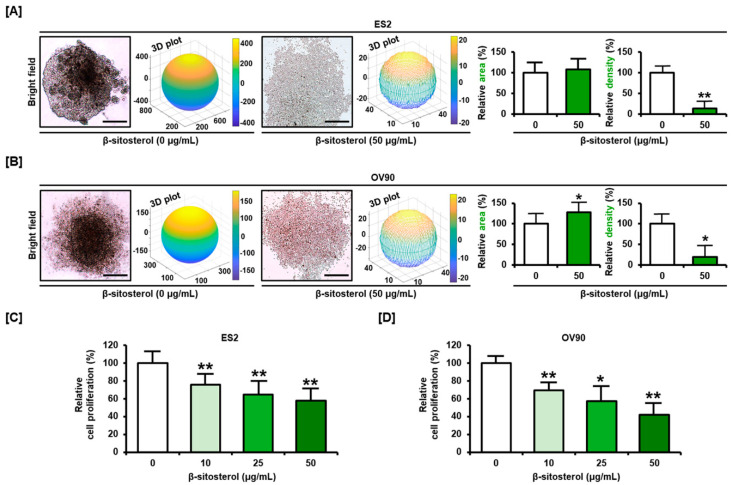
Restriction of cell growth by β-sitosterol in ovarian cancer cells. (**A**,**B**) Analysis of 3D spheroid formation by ES2 (**A**) and OV90 (**B**) cells with or without β-sitosterol. (**C**,**D**) Cell proliferation assay following β-sitosterol treatment (0, 10, 25, and 50 µg/mL) for both cell lines. Comparative bar graphs represent the % of cell growth compared to the vehicle-treated control (100%) for ES2 (**C**) and OV90 cells (**D**). The asterisks indicate significant differences between treated and control cells (** *p* < 0.01, and * *p* < 0.05).

β-sitosterol inhibited the growth of both cell lines. β-sitosterol reduced 3D spheroid formation by these ovarian cancer cells with low ability of aggregation (Figure 5A,B). The proliferation of ES2 cells (Figure 5C) was decreased to 61.0%, and that of OV90 cells (Figure 5D) to 54.0% by 50 µg/mL of β-sitosterol.

### 3.6. Inactivated Intracellular Signals Caused by β-Sitosterol in Ovarian Cancer Cells

**Figure 6:**
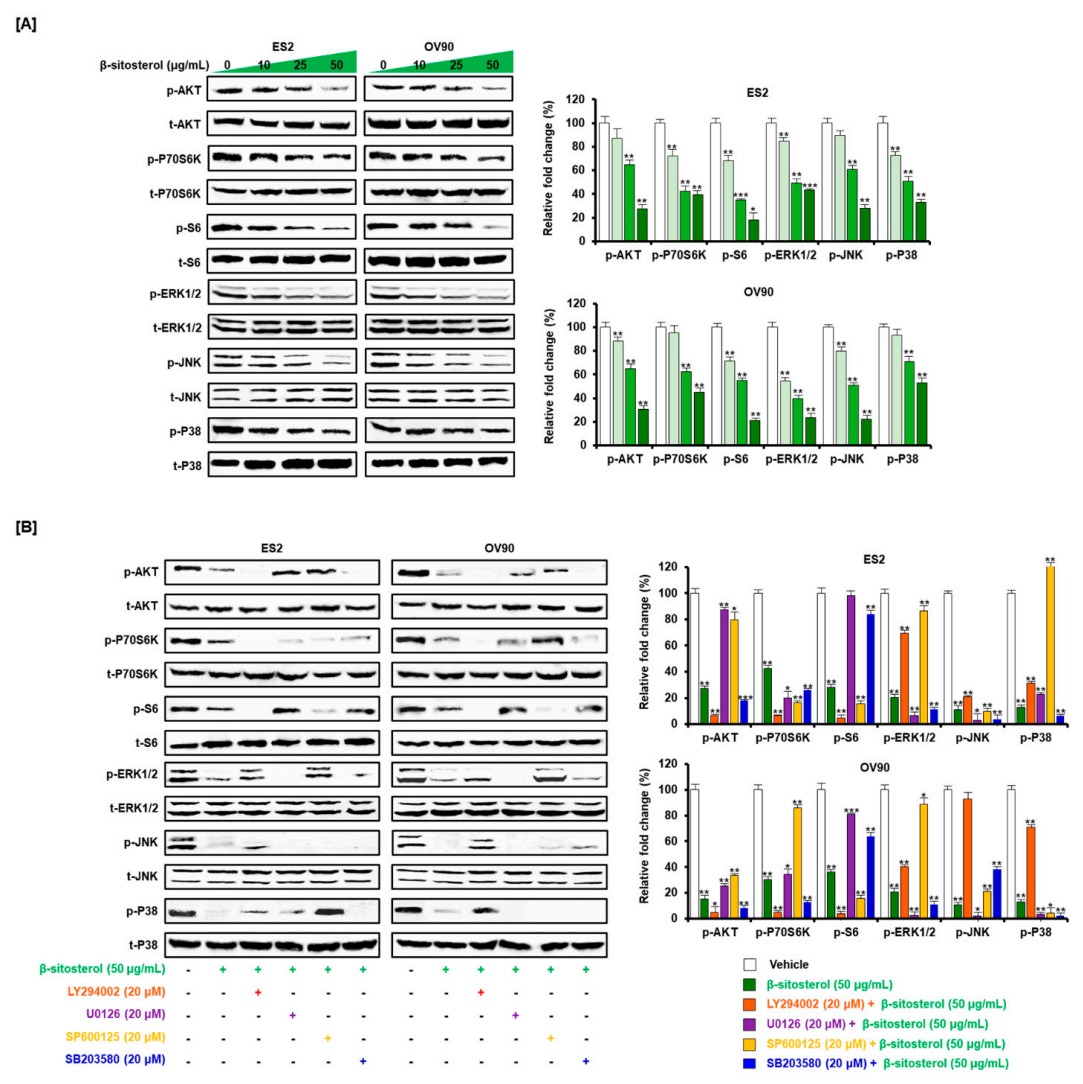
Change of cell growth-related signals by β-sitosterol in the two cell lines. (**A**) Western blots show phosphorylation changes in the PI3K pathway including protein kinase B (AKT), P70S6 kinase (P70S6K), S6 and in the MAPK pathway including extracellular signal-regulated kinase 1/2 (ERK1/2), c-Jun N-terminal kinase (JNK), P38, in ovarian cancer cells after β-sitosterol (0, 10, 25, and 50 µg/mL) treatment. (**B**) Western blots show changes in the phosphorylation levels of proteins in the PI3K and MAPK signaling pathways in ovarian cancer cells following co-treatment with β-sitosterol and each inhibitor. The asterisks indicate significant differences between treated and control cells (*** *p* < 0.001, ** *p* < 0.01, and * *p* < 0.05).

Cell proliferation-related intracellular signaling pathways were identified using western blot analysis. The proportion of the examined proteins showing phosphorylation (AKT, P70S6K, and S6) was found to be decreased by treatment with β-sitosterol (0, 10, 25, and 50 µg/mL; Figure 6A). Moreover, the phosphorylation of ERK1/2, JNK, and P38 was inhibited by β-sitosterol (0, 10, 25, and 50 µg/mL) in a dose-dependent manner, when compared to the total amount of each protein in the cells. Next, we observed a more detailed signal correlation using signal caspase inhibitors, including a PI3K inhibitor (LY294002; 20 µM), an ERK1/2 inhibitor (U0126; 20 µM), a JNK inhibitor (SP600125; 20 µM), and a P38 inhibitor (SB203580; 20 µM). The cells were treated with each inhibitor prior to β-sitosterol treatment (Figure 6B). The phosphorylation of AKT was almost completely inhibited by LY294002 and SB203580 in ES2 and OV90 cells. The phosphorylation of P70S6K was almost blocked by LY294002, U0126, SP600125, and SB203580 in ES2 cells and by LY294002 and SB203580 in OV90 cells. The phosphorylation of S6 was decreased by all inhibitors except U0126 in ES2 cells and by all inhibitors except U0126 and SP600125 in OV90 cells. The phosphorylation of ERK1/2 was completely inhibited by U0126 and SB203580 in both cell lines and slightly decreased by LY294002 in ES2 cells. The phosphorylation of JNK was completely blocked by all inhibitors. The phosphorylation of P38 was completely blocked by all inhibitors except SP600125 in ES2 cells.

### 3.7. Suppression of Cell Migration and Enhancement of the Anti-Proliferation Effect of Existing Conventional Chemotherapeutic Agents by β-Sitosterol

**Figure 7:**
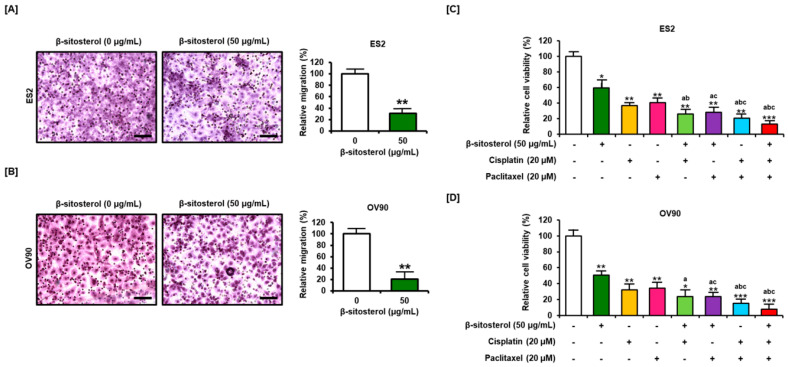
Anti-migration and -proliferation effects of β-sitosterol with or without anticancer drugs. (**A**,**B**) Migration of ovarian cancer cells following treatment with β-sitosterol in ES2 (**A**) and OV90 (**B**) cells. (**C**,**D**) Enhancement of the anti-proliferation effect of existing conventional chemotherapeutic agents by β-sitosterol. Bar graphs show the viability (percentage) of ovarian cancer cells following treatment with combinations of β-sitosterol and cisplatin or paclitaxel. Data are shown relative to the vehicle-treated controls (100%). The asterisks indicate significant differences between treated and control cells (*** *p* < 0.001, ** *p* < 0.01, and * *p* < 0.05). Lower-case letters indicate significant differences within the group as follows: ‘a’ compared to β-sitosterol, ‘b’ compared to cisplatin, and ‘c’ compared to paclitaxel.

The migration ability of ES2 and OV90 cells was estimated to decrease by 30.5% and 20.5%, respectively, following treatment with 50 µg/mL of β-sitosterol as compared to vehicle-treated cells (Figure 7A,B). Cell proliferation assays were performed to determine whether β-sitosterol has synergistic effects with existing anticancer drugs (cisplatin and paclitaxel). The two cell types were treated with β-sitosterol alone or in combination with cisplatin or paclitaxel. β-sitosterol was found to further increase cisplatin- and paclitaxel-induced restriction of the growth of human ovarian cancer cells (Figure 7C,D).

## 4. Discussion

Phytosterols are found in a various plants and have long been part of our diets. β-sitosterol has been reported to have various physiological activities [6,7,8]. In addition, it has been reported to have anticancer effects; for example, β-sitosterol impairs the development of human colon cancer (HT-29) cells by stimulating the sphingomyelin cycle [8] and suppresses the growth of both the androgen-dependent prostate cancer cell line LNCaP [25] and the androgen-independent prostate cancer cell line PC-3 [26]. The differentiation ability of the human cancer cells A549 is decreased by β-sitosterol, which also induces G0/G1 cell cycle arrest, decreased CDK4 and cyclin D1 levels, and increased expression of p21/Cip1 and p27/Kip1 [27]. In colon cancer, β-sitosterol decreases the expression of proliferating cell nuclear antigen (PCNA), which is known as an indicator of cells’ proliferative activity [28,29]. Similarly, in our experiments, β-sitosterol inhibited the proliferation of human ovarian cancer cells ES2 and OV90. In other studies, β-sitosterol caused apoptosis, induced the activation of caspases in human breast cancer MDA-MB-231 cells, and also activated FAS signaling [30]. FAS is a member of the tumor necrosis factor receptor family that plays a central role in programmed cell apoptosis and has been implicated in the development of various malignancies [30]. β-sitosterol reduced the expression of both Bcl-2 and “cellular inhibitor of apoptosis protein-1” (cIAP1), while inducing the activation of BAX and cytochrome C in the human colon cancer cells HT116 [11]. Moreover, β-sitosterol caused the activation of caspase-3 and BAX in the lymphoma cells U937 [31]. Likewise, we identified that β-sitosterol induced late apoptosis via the activation of apoptotic signals, including cleaved caspase 3 and caspase 9, cytochrome C, BAX, and BAK, in human ovarian cancer.

Mitochondrial dysfunction and destruction leads to the activation of pro-apoptotic proteins and cell death [32]. The regulation of intracellular calcium levels by the mitochondria regulates cell fate; an overload of calcium can induce programed cell death [33,34]. We found that β-sitosterol dose-dependently increased calcium concentrations in the cytoplasm and mitochondria in human ovarian cancer cells. ROS is essential for cell survival, but excessive ROS generation leads to cell death. Excessive ROS levels in the cell induce cell death initiated by intrinsic apoptotic signals in the mitochondria or extrinsic apoptotic signals by death receptor pathways. In the intrinsic pathway, ROS cause mitochondrial membrane depolarization and release AIF, Endo G, cytochrome C, and Smac/Diablo into the cytosol by opening BAX/BAK channels on the outer mitochondrial membrane. Cytochrome C then forms the apoptosome complex in the cytosol with Apaf-1 and procaspase-9, leading to the activation of caspase-9, which subsequently induces the cleavage of caspase-3, resulting in apoptosis-induced cell death. ROS directly damage nuclear and mitochondrial DNA [35,36]. Moreover, excessive levels of ROS disturb ER protein folding and induce ER stress and UPR, leading to disruption of cell homeostasis and apoptosis [37]. Cisplatin, a representative anticancer agent for ovarian cancer, induces cytotoxicity via increased ROS production [38]. According to a report by Awad et al. (2005), β-sitosterol promoted ROS generation in the prostate cancer cells PC-3 [14]. In our study, β-sitosterol also induced an increase in ROS production in the two ovarian cancer cell lines examined. These results indicated that β-sitosterol stimulates oxidative stress and changes in calcium homeostasis leading to mitochondrial dysfunction and cell death of ovarian cancer cells.

The ER is an important organelle that produces and processes proteins necessary for cell survival and regulates intracellular calcium dynamics [39]. Disruption of ER homeostasis leads to ER stress, accompanied by the activation of the unfolded protein response (UPR), which can initiate apoptosis in cancer cells [40]. In addition, accumulation of calcium released from the ER can be caused by IP3R-dependent mechanisms involving changes in ER-mitochondria tethering proteins. In addition, the complex VAPB-FAM82A2 supports ER-mitochondria contact for calcium delivery and regulates autophagy, leading to death of diverse cancer cells [18,41,42]. Therefore, miscommunication between ER and mitochondria causes an impairment of cellular functions through calcium. Furthermore, the ROS scavenger N-acetylcysteine decreased ER stress proteins and protected the mitochondria from oxidative stress, thus mitigating cell death [43,44]. Likewise, many previous studies have shown a clear association between ROS production and mitochondria-related cell death. Similar to previous studies, we found that β-sitosterol induced the activation of the UPR and the ER-mitochondrial axis with increased cytosolic and mitochondrial calcium ion levels in human ovarian cancer cells.

Cancer cells often present the activation of intracellular proliferation-related signaling pathways; therefore, various anticancer drugs work by inhibiting PI3K and MAPK pathways [45,46,47,48]. Reduction of Akt was observed in β-sitosterol-treated murine fibrosarcoma cells (MCA-102) [10], and we found that β-sitosterol inhibited the PI3K and MAPK signaling pathways in ES2 and OV90 cells. β-sitosterol has also been reported to decrease the metastatic ability of human breast cancer MDA-MB-231 cells [12]; similarly, we observed a decrease in the migration ability of ovarian cancer cells. In particular, β-sitosterol has a synergistic anticancer effect when administered with tamoxifen in breast cancer MCF-7 and MDA-MB-231 cells [15]; this was comparable to the effects observed in this study following treatment of ovarian cancer cells with combinations of β-sitosterol and cisplatin or paclitaxel. Compared to treatments with only β-sitosterol, cisplatin, or paclitaxel, the combined treatment of β-sitosterol with cisplatin or paclitaxel significantly suppressed the proliferation of human ovarian cancer cells.

## 5. Conclusions

β-sitosterol caused late cell apoptosis, together with the activation of pro-apoptotic signals, in human ovarian cancer cells ES2 and OV90. These findings are supported by observations of the loss of MMP and the accumulation of calcium in the cytoplasm and mitochondria. ROS production and ER stress were also increased by β-sitosterol in both cell lines. In addition, β-sitosterol activated the ER-mitochondrial axis in ovarian cancer cells, while cell growth and the cell cycle were inhibited in a dose-dependent manner, and PI3K/MAPK signal transduction was decreased. In particular, β-sitosterol further increased ovarian cancer cell death when used in combination with cisplatin or paclitaxel. The results of this study suggest that β-sitosterol can be used as a novel anticancer agent to treat human ovarian cancer.

## Data Availability

Data are contained within the article.
